# Adenoviral delivery of soluble ovine OX40L or CD70 costimulatory molecules improves adaptive immune responses to a model antigen in sheep

**DOI:** 10.3389/fcimb.2022.1010873

**Published:** 2022-09-23

**Authors:** José M. Rojas, Carolina Mancho, Andrés Louloudes-Lázaro, Daniel Rodríguez-Martín, Miguel Avia, Santiago Moreno, Noemí Sevilla, Verónica Martín

**Affiliations:** ^1^ Centro de Investigación en Sanidad Animal, Instituto Nacional de Investigación y Tecnología Agraria y Alimentaria, Consejo Superior de Investigaciones Científicas (CISA-INIA-CSIC), Madrid, Spain; ^2^ Departamento de Investigación Agroambiental, Instituto Madrileño de Investigación y Desarrollo Rural, Agrario y Alimentario (IMIDRA), Madrid, Spain; ^3^ Departamento de Producción Animal, Instituto Madrileño de Investigación y Desarrollo Rural, Agrario y Alimentario (IMIDRA), Madrid, Spain

**Keywords:** immune response, CD70, OX40L, adenoviral vectors, vaccine

## Abstract

The tumour necrosis factor superfamily OX40L and CD70 and their receptors are costimulatory signalling axes critical for adequate T and B cell activation in humans and mice. In this work we inoculated groups of sheep with human recombinant adenovirus type 5 (Ad) expressing *Ovis aries* (*Oa)*OX40L or *Oa*CD70 or a control adenoviral vector to determine whether they could improve the immune response to the model antigen OVA. PBMCs and serum samples were obtained for analysis of the adaptive immune response to OVA at days 0, 15, 30 and 90 post-inoculation (pi). Recall responses to OVA were assessed at day 7 and 30 after the second antigen inoculation (pb) at day 90. Administration of these immunomodulatory molecules did not induce unspecific PBMC stimulation. While *Oa*OX40L administration mainly increased TNF-α and IL-4 in PBMC at day 15 pi concomitantly with a slight increase in antibody titer and the number of IFN-γ producing cells, we detected greater effects on adaptive immunity after *Oa*CD70 administration. Ad*Oa*CD70 inoculation improved antibody titers to OVA at days 30 and 90 pi, and increased anti-OVA-specific IgG-secreting B cell counts when compared to control. Moreover, higher IFN-γ production was detected on days 7 pi, 7 pb and 30 pb in PBMCs from this group. Phenotypic analysis of T cell activation showed an increase in effector CD8^+^ T cells (CD8^+^ CD62L^-^ CD27^-^) at day 15 pi in Ad*Oa*CD70 group, concurrent with a decrease in early activated cells (CD8^+^ CD62L^-^ CD27^+^). Moreover, recall anti-OVA CD8^+^ T cell responses were increased at 7 pb in the Ad*Oa*CD70 group. Ad*Oa*CD70 administration could therefore promote CD8^+^ T cell effector differentiation and long-term activity. In this work we characterized the *in vivo* adjuvant potential on the humoral and cellular immune response of *Oa*OX40L and *Oa*CD70 delivered by non-replicative adenovirus vectors using the model antigen OVA. We present data highlighting the potency of these molecules as veterinary vaccine adjuvant.

## Introduction

The ultimate purpose of vaccination is to generate potent and long-lasting protection against diseases. Traditional vaccination approaches were often considered to be successful in controlling pathogens based on their capacity to induce neutralizing antibodies. There is nonetheless now increasing evidence that activation of T cells is a requisite to achieve long-term success for any vaccine. Ideally, T cell activation with viral antigens should promote a robust expansion of naïve precursor T cell, their differentiation to effector cells, and the survival over time of these virus-specific effector cells as high frequency memory T cells so that optimal surveillance against subsequent infections is achieved. T cells, in addition to carrying out effector mechanisms such as cytotoxicity, modulate the immune response through helper mechanisms that aim to eradicate the pathogen. Optimal T cell activation is a complex process that involves antigen recognition on MHC molecules and secondary costimulatory signals provided by different molecules (ligands) that drive the activation and survival of the antigen-specific T cell (Lafferty et al., 1983; Mueller et al., 1989). Thus, the use of immunostimulatory ligands in combination with a vaccine antigen has the potential to improve vaccination.

Receptors and ligands of the tumor necrosis factor (TNF) family are key in controlling co-stimulation of many types of immune reactions regulated by T cell help (Croft, 2003), like memory cell differentiation or antibody secreting plasma cell activation (Bishop and Hostager, 2001). Costimulatory interactions between members of the TNF receptor (R)/TNF superfamily, such as those between CD27/CD70 or OX40/OX40L, are involved in these processes, by directing lymphocyte activation, proliferation and maintenance during antiviral immune response (Duttagupta et al., 2009; Wortzman et al., 2013). These TNFR/TNF pairings could thereby be targeted to promote cellular immune responses induced by vaccination.

OX40L is expressed on the surface of antigen-presenting cells (Godfrey et al., 1994; Al-Shamkhani et al., 1996), such as conventional dendritic cells (DC) (Ohshima et al., 1997), 24-48 hours upon exposure to thymic stromal lymphopoietin, CD40L or Toll-like receptor (TLR) agonists (Ito et al., 2005; Krause et al., 2009). OX40L signals after engagement with its receptor, OX40 (also known as CD134 or TNFRSF4), which is predominantly, but not exclusively, expressed on activated T cells. This interaction controls the robust expansion of naïve precursor T cells, their differentiation to effector cells and their survival as memory cells (Fu et al., 2020). After this first interaction, the OX40-expressing activated T cells can abandon the DCs and interact with other OX40L-expressing cells during the effector phase of the immune response, such as activated CD4^+^ T cells (Soroosh et al., 2006), B cells (Linton et al., 2003), nature killer (NK) cells, NKT cells, neutrophils, monocytes, myocytes, endothelial cells or mast cells provoking a T cell-mediated multi-channel effector activation. Binding of OX40L results in OX40 trimerization, recruitment of TRAF 2, 3 and 5, and signaling through the NF-κB pathway, which allows entry of RelA and p50 into the nucleus. This pairing signaling also participates in the T cell receptor activation *via* the phosphatydil-inositol-3 kinase (PI3K) pathways (Weinberg et al., 2004; Croft, 2010). The OX40/OX40L pairing is not only directly engaged in T cell expansion and survival, but also determines indirectly the pool of cytokines produced, thus generating a bias in the T response that is dependent on the context. Furthermore, this costimulatory axis also activates CD8^+^ T cell during viral infections and alters the activity and differentiation of regulatory T cells (Treg).

The canonical and the alternative NF-κB pathways are also activated by the interaction of another pair of TNF superfamily molecules (Akiba et al., 1998), CD70/CD27. The ligand CD70 (also named TNFSF7) (Grant et al., 2017), transiently expressed on mature dendritic cells (mDC), antigen-activated B and T cells and NK cells, signals through its receptor, CD27 constitutively expressed on naïve T, γδ T cells and memory B and T cell populations and subsets of NK-cells. The constitutive CD27 expression in naïve T cells suggests an earlier turn-on during priming processes than the OX40 signaling that requires TCR stimulation to be expressed (1998; Gramaglia et al., 2000; Rogers et al., 2001; Bansal-Pakala et al., 2004). CD27 activation is important for the progress of long-lasting adaptive immunity, as it is involved in promoting plasma B cell formation, IgG production and differentiation of memory cell through T cell activation (Kobata et al., 1995). CD27 signaling is also important for the expansion of antigen-specific naïve T cells and their maintenance as memory cells (Hendriks et al., 2000).

The use of these TNF/TNFR as adjuvants in classical vaccination has been proposed due to their involvement in immune responses against viral infections (Duttagupta et al., 2009; Gupta et al., 2013). In a murine HIV-1 DNA vaccine model, secreted forms of OX40L and CD70 enhanced cellular and humoral immune responses (Kanagavelu et al., 2012). Engagement of soluble ligand forms of OX40L and CD70 on activated lymphocytes has therefore the potential to improve antigen specific T and B cell responses.

Most of the important diseases that affect ruminants such as foot and mouth disease, peste des petits ruminants, bluetongue, or zoonotic diseases like rift valley fever (Diaz-San Segundo et al., 2017; Faburay et al., 2017; Kumar et al., 2017; van Rijn, 2019), are identified as targets for vaccination. Improving current vaccination procedures could therefore be beneficial to control these diseases. We have previously characterized the activity and role of the OX40/OX40L and CD27/CD70 signaling axes in sheep. Our data showed that ovine CD27 is expressed on the majority of circulating CD4^+^ and CD8^+^ T cells and on a subset of CD335^+^ NK cells, while being present at low levels on circulating B cells (Rojas et al., 2020). Additionally, we generated two recombinant adenoviruses, AdOX40L and AdCD70 expressing ovine [*Ovis aries* (*Oa*)] OX40L and CD70 soluble molecules, respectively, that allow the activation of these costimulatory signaling routes of TNFR superfamily in sheep (Rojas et al., 2020). The capacity of soluble ovine OX40L and CD70 forms to activate cells expressing their cognate receptor and stimulate ovine T cell responses was demonstrated in culture cells, pointing to their potential use as adjuvant for vaccination strategies in ruminants.

Adenoviral vectors are effective foreign gene delivery vehicles *in vivo* (Crystal, 2014; Rojas et al., 2014a; Martín et al., 2015). Recombinant non-replicative human adenoviruses are specially indicated for use in the veterinary field as they avoid problems derived from the preexisting host immunity. Furthermore, these vectors have several advantages such as low toxicity, safety, easy handling and distribution, and the capacity to transduce wide-ranging host species (Rojas et al., 2019).

In this work we characterized the *in vivo* adjuvant potential on the humoral and cellular immune response of *Oa*OX40L and *Oa*CD70 delivered by non-replicative adenovirus vectors using the model antigen OVA. We present data highlighting the potency of these molecules as veterinary vaccine adjuvant.

## Materials and methods

### Cells and viruses

Vero cells (ATCC CCL-81) and HEK293 cells (ATCC CRL-1573) were grown in Dulbecco’ minimal essential medium (DMEM, Gibco, Dublin, Ireland), supplemented with 10% Foetal Bovine Serum (FBS) (Sigma-Aldrich, Saint Louis, MO, USA), 2 mM L-glutamine, 1%100x non-essential amino-acids (AANE), 1 mM sodium pyruvate and 100 U/mL Penicillin/100 μg/mL Streptomycin (all from Thermofisher Scientific, Waltham, MA, USA). Final stocks of the recombinant adenoviruses AdOX40L, AdCD70 and AdDsRed were purified and titrated after sequential rounds of growth on HEK293 cells using standard protocols as described before (Rojas et al., 2020) and stored at -70°C until used.

The procedure to obtain recombinant adenoviruses has been described in detail before (Rojas et al., 2020).

### Experimental design

Three groups of 7 one-year old female “*Rubia del Molar*” breed sheep (21 sheep in total) were inoculated intramuscularly with 10^8^ infectious units (IU) of recombinant adenovirus expressing *Oa*OX40L or *Oa*CD70, or the control adenoviral vector AdDsRed, respectively, at the time of immunization with 20 mg/sheep OVA and bled at days 0, 15, 30 and 90 post-inoculation (D0, D15, D30, 3MD0). Recall responses to OVA were assessed by inoculating the same antigen amount three months later (3MD0), and blood samples obtained at days 7 and 30 after the second OVA inoculation (3MD7 and 3MD30). PBMCs and serum samples were obtained for analysis of the adaptive immunity induced to OVA and obtained as described in (Rojas et al., 2014b; Rodríguez-Martín et al., 2021). The sheep were housed in the Experimental Farm “La Chimenea” (IMIDRA) in excellent animal welfare conditions. No abnormal signs were observed in any animal during the experimental time.

### OVA specific antibodies detection

#### Specific Anti-OVA IgG and IgM ELISA detection

Antibodies against OVA were detected using ELISA plates (Maxisorp; Nunc) coated for 1 h at room temperature with 100 μL/well of 20 μg/mL OVA (Sigma-Aldrich, Saint Louis, MO, USA) in 0.1 M carbonate buffer (pH 9.6). Serial dilutions of sera from inoculated sheep were added to the plates and incubated for 1 h at room temperature, after blocking with blocking buffer (PBS with 0,05% (vol/vol) Tween-20 and 4% (wt/vol) skimmed milk) for 1 h at room temperature and washing five times with 1% (vol/vol) Tween-20 in PBS. The presence of OVA-specific IgG or IgM was detected using a secondary donkey anti-sheep IgG conjugated to HRP (1/2,000; Serotec, Corston Bath, UK) or rabbit anti-sheep IgM also conjugated to HRP (1/20,000; Bethyl, Montgomery, Texas, USA). After washing 10 times with 1% (vol/vol) Tween-20 in PBS, signal was developed using TMB Liquid Substrate System (Sigma-Aldrich, Saint Louis, MO, USA) and the reaction was stopped with 3 M sulfuric acid before reading. OD was determined at 450 nm on a FLUOstar Omega (BMG Labtech, Ortenberg, Germany) ELISA plate reader. All IgG/IgM measurements were made in triplicate, and assays were only considered valid when SDs were below 10% of the average. IgG/IgM binding to OVA was considered positive only when the OD obtained was at least twice the OD obtained with the preimmune serum from the same sheep.

#### Quantification of anti–OVA-specific IgG-secreting B cells by ELISPOT

MSIPS4510 plates (Millipore, Merck, Darmstadt, Germany) were activated using sterile 35% (vol/vol) ethanol for 1 min, and after thorough washing with sterile water, incubated overnight at 4°C with 20 μg/mL OVA (Sigma-Aldrich, Saint Louis, MO, USA) in 0.1 M carbonate buffer, pH 9.6. Plates were blocked with PBS containing 4% (wt/vol) dried skimmed milk for 2 h at 37°C. Fresh PBMCs were suspended at 5x10^6^ cells/mL, and 1:2 dilutions were performed in RPMI medium (supplemented with L-glutamine, 1 mM sodium pyruvate, 25 mM Hepes, nonessential amino acids, and 10% (vol/vol) inactivated FBS) down to a concentration of 1x10^5^ cells/mL; 100 μL/well of each cell suspension (in triplicate) was incubated overnight at 37°C, 5% (vol/vol) CO_2_. After discarding the cells and washing with PBS, membranes were incubated with anti-sheep IgG conjugated to HRP (Serotec, Corston Bath, UK) for 3 h at room temperature. After five washes in PBS, membranes were developed using 3,3′,5,5′- tetramethylbemzidine (TMB) substrate (Mabtech, Sweden). Once spots were formed, membrane were washed with abundant distilled water and allowed to dry in the dark. Results were expressed as the number of antigen-specific cells (ASCs) per 10^6^ PBMCs.

### Detection of specific IFN-γ T cell secretion against OVA by ELISPOT

Ovine IFN-γ ELISPOT assays were performed using MSIPS4510 plates (Millipore, Merck, Darmstadt, Germany). Membranes were activated using sterile 35% ethanol for 1 min, and after thorough washing with sterile water, incubated overnight at 4°C with 5 μg/ml anti-bovine IFN-γ antibody (MT17.1, Mabtech, Sweden). Plates were blocked in RPMI (supplemented with glutamine, Na+ -pyruvate, HEPES, non-essential amino acids, antibiotics and 10% FBS) for 2 h at room temperature. Sheep PBMCs were then plated at a density of 2–3x10^5^ cells per well and incubated with 20μg/mL OVA, PBMC medium as negative control or Concanavalin- A (Con-A) (1.25 μg/ml) as positive control for 48 h at 37°C, 5% CO2. After discarding the cells and washing with PBS, membranes were incubated with biotin-labelled anti-bovine IFN-γ antibody (MT307-biotin, Mabtech, Sweden) diluted at 0.25 μg/ml in PBS +0.5% FCS for 2 h. After 5 washes in PBS, membranes were incubated for 1 h with streptavidin conjugated to alkaline phosphatase (ExtrAvidin-AP, Sigma-Aldrich, Saint Louis, MO, USA) diluted 1:10,000 in PBS+ 0.5% FBS. Membranes were washed thoroughly first in PBS and then in distilled water before ELISPOT assay reactions were developed using Sigma FAST BCIP/NBT (Sigma-Aldrich, Saint Louis, MO, USA). Once spots were formed, membranes were washed with abundant distilled water and allowed to air dry in the dark. All cultures were performed in triplicates and ELISPOT assays were considered valid only when average spot counts were below 25 for control cultures and standard deviations in positive wells below 15% of the average counts.

### Intracellular cytokine staining and flow cytometry

To characterize PBMC populations during the course of the experiment, the following antibodies were used: anti-ovine CD4 (clone 44.38), CD8 (clone 38.65), and WC1 (clone 19.19) (all from Biorad, Madrid, Spain); anti-human CD14 (clone TÜK4), CD16 (clone KD1) (both from Biorad, Madrid, Spain) and CD27 (clone LG3.A10) (Biolegend, San Diego, CA, USA); and anti-bovine B cell marker (clone BAQ44A) (KingFisher Biotech, Minnesota, USA) and CD62L (clone CC32) (Biorad, Madrid, Spain). Cells were labeled as described in (Rodríguez-Martín et al., 2021). To assess IFN-γ production, PBMC were left unstimulated as control or stimulated with 10μg/ml OVA for 6 hours. As positive control, PBMC were stimulated with 1.25 μg/mL concanavalin-A (ConA) (Sigma-Aldrich, Saint Louis, MO, USA) for 3 hours. Brefeldin-A (5μg/mL) (Biolegend, San Diego, CA, USA) was added in the last 3 h of incubation. Cells were then labelled as described in (Rojas et al., 2014b; Rojas et al., 2017b) with anti-sheep-CD4-FITC (clone 44.38), anti-sheep-CD8-PE (clone 38.65), and anti-bovine-IFN-γ-Alexa 647 (clone CC302) antibodies (all from Bio-Rad, Madrid, Spain). The BD Cytofix/Cytoperm kit (BD Biosciences, NJ, USA) was used for cell fixation and permeabilization according to the manufacturer’s protocol. Dead cells were excluded from the analysis by staining with the Live-Dead Fixable Near-IR (Thermofisher Scientific, Waltham, MA, USA) viability marker. Samples were acquired on a FACSCalibur or a FACSCelestaSorp flow cytometer (Becton Dickinson, NJ, USA) and data analyzed with FlowJo software (Tree Star Inc.). Percentage of IFN-γ^+^ cells within the CD4^+^ or CD8^+^ T cell compartment was measured and data presented as IFN-γ production above the background of unstimulated cells (net IFN-γ). Gating strategy for IFN-γ^+^ events is detailed in **Supplementary Figure 1**. Isotype and fluorescence minus one-channel controls were used for gating strategy.

### RNA extraction and qRT-PCR

PBMCs RNA was extracted using TRIzol Reagent Solution (Thermofisher Scientific, Waltham, MA, USA) and cDNA generated by reverse transcription using oligo dT primers with SuperScript III reverse transcriptase (Invitrogen, Thermofisher Scientific, Waltham, MA, USA). Transcription levels of TNF-α, IL-12, IL-10, IL-1β, IL-4, IL-6, and IL-2 were evaluated by real-time PCRs performed in a Light Cycler 480 System instrument (Roche, Merck, Darmstadt, Germany) in a Light Cycler 480 SYBR Green I Master Reagents (Roche, Merck, Darmstadt, Germany) using primers and RT-qPCR conditions detailed in **Supplementary Table 1**. Gene expression was normalized to β-actin RNA gene expression, and relative expression levels were calculated using the 2^-ΔΔCT^ method (Winer et al., 1999; Schmittgen et al., 2000). A melting curve for each PCR fluorescence reading, every degree between 60 and 95°C, was determined to ensure that only a single product had been amplified.

### Statistical analyses

Data handling and statistical analyses was performed using Prism 6.0 software (GraphPad Software Inc. San Diego, CA, USA). Statistical tests used to compare data are indicated in the figure legends.

## Results

### Characterisation of PBMCs

**Figure 1:**
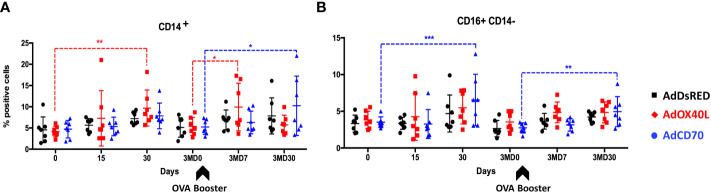
A slight increase in peripheral monocytes and NK cells is detected after AdOX40L and AdCD70 administration. AdDsRed, AdOX40L or AdCD70 were administered at the time of immunization with OVA in three different groups of sheep. PBMC were obtained at different timepoints: day 0 (immunization), D15, D30, 3MD0 (prior to booster inoculation with OVA), 3MD7 (i.e. 7 days post-booster), and 3MD30 (i.e. 30 days post-booster); and percentages of **(A)** CD14+ (monocytes), and **(B)** CD16+ CD14- (NK cells) PBMC evaluated by flow cytometry. Arrowheads denote OVA booster inoculations at day 90 (3MD0). *p < 0.05; **p < 0.01; ***p < 0.001 (two-way ANOVA with Dunnett’s post test).

To determine whether delivery of these costimulatory molecules could produce unspecific expansion of PBMC populations, we assessed by flow cytometry the changes in percentage of CD4^+^, CD8^+^, WC1^+^, B cell marker^+^, CD14^+^ and CD14^-^ CD16^+^ (NK cells) cells in PBMCs obtained from the three inoculated sheep groups over the course of the experiment. AdOX40L and AdCD70 administration does not trigger unspecific PBMC expansion in CD4^+^, CD8^+^, WC1^+^ or B cell marker^+^ populations (**Supplementary Figure 2**). Nevertheless, CD14^+^ cells (monocytes) (**Figure 1A**) significantly increased after antigen exposure in the AdOX40L group at day 30 and at day 7 post-booster (3MD7), while a significant increase in monocytes was also detected at 3MD30 in the AdCD70 group when compared to baseline (D0 or 3MD0). We also noted a significant increase in CD16^+^CD14^-^ cells (putatively NK cells) in the AdCD70 group 30 days after antigen exposure at D30 and 3MD30 (**Figure 1B**). In spite of these differences in monocytes and NK cells, AdCD70 and AdOX40L administration did not appear to induce drastic unspecific stimulation of lymphocytes.

**Figure 2:**
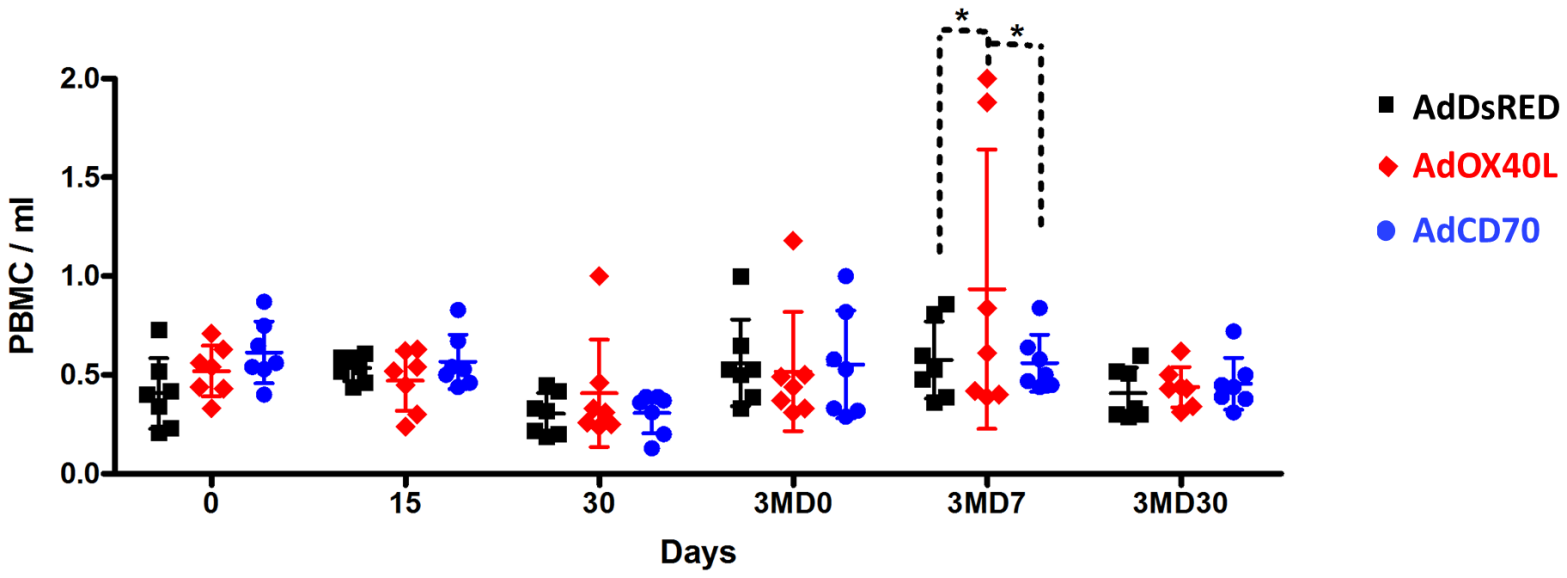
AdOX40L or AdCD70 did not produce unspecific PBMC expansion. PBMC were isolated at day 0, 15, 30, 90 (pre-boost with OVA), 3MD7 (7 days post-boost), and 3MD30 (30 days post boost) and counted. PBMC/ml of blood are plotted for each OVA immunization group (AdDsRed, Ad*Oa*OX40L or Ad*Oa*CD70). *p < 0.05 two-way ANOVA with Bonferroni’s post-test.

This was further confirmed when we assessed PBMC numbers in sheep that received AdOX40L or AdCD70 inoculations. PBMC numbers were similar in all three groups indicating that AdOX40L or AdCD70 did not induce unspecific PBMC expansion. It is noteworthy that an increase in total PBMC was observed in 2 sheep that received AdOX40L after OVA booster inoculation at 3MD7, which could represent the expansion of anti-OVA lymphocytes (**Figure 2**). Overall AdOX40L or AdCD70 inoculation appears safe, as no indication of unspecific lymphocyte expansion occurred *in vivo* and the sheep did not show any adverse clinical sign to the administration throughout the experiment.

### *Oa*CD70 increased specific antibodies production against OVA

**Figure 3:**
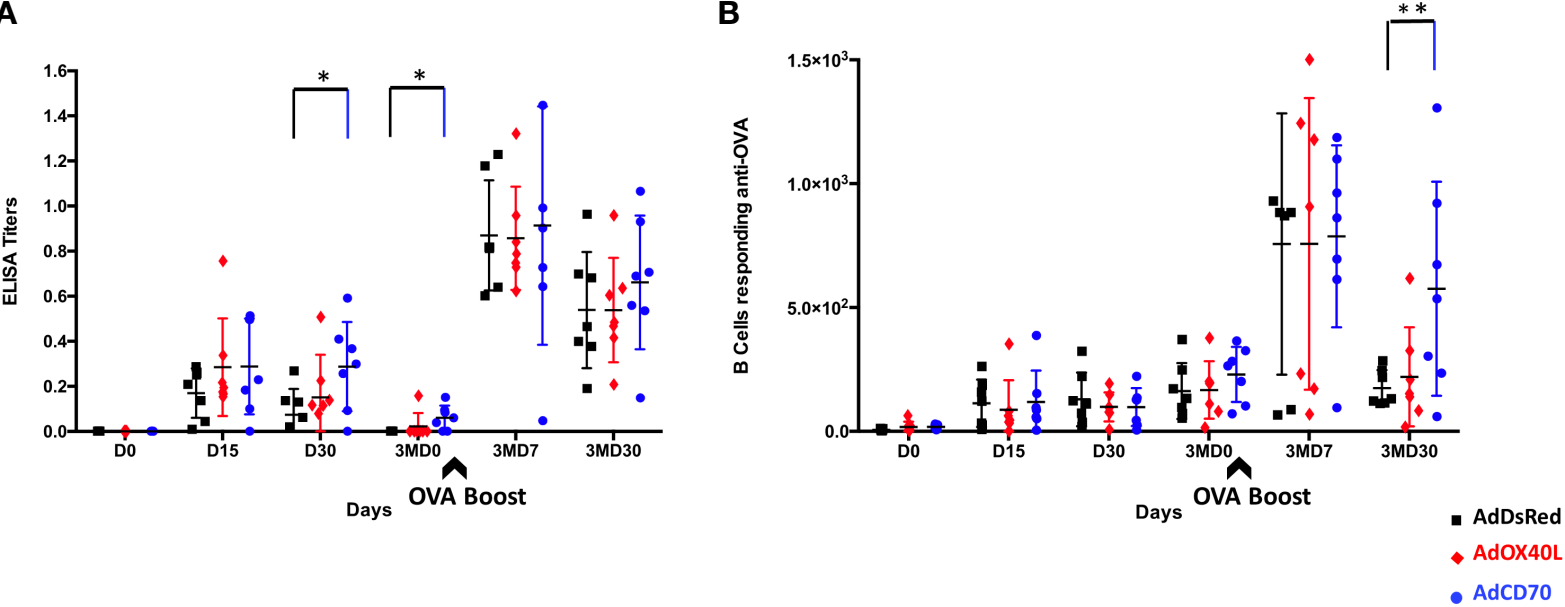
OVA specific antibodies production in inoculated sheep. **(A)** Detection of anti-OVA-specific antibodies by ELISA assay. Inoculation with OVA concomitantly with recombinant adenovirus expressing the *Oa*OX40L or *Oa*CD70 proteins induces OVA-specific IgG in sheep. Groups (n = 7) of sheep were inoculated intramuscularly with OVA simultaneously with control vector AdDsRed (black), AdOX40L (red), or AdCD70 (blue), respectively, at day 0; and 3 months later (3MD0) boosted only with OVA (indicated by the arrow). At the indicated time points, the serum samples obtained were analyzed for OVA-specific IgG by ELISA using OVA-coated plates. Data are presented as OD read at 450nm IgG titer for each animal. *p < 0.05 days 30 and 3MD0 AdCD70 inoculated sheep vs. AdDsRed inoculated sheep the same days, respectively (one-way ANOVA). **(B)** Detection of anti-OVA-specific IgGs-secreting B cells by ELISPOT assay. PBMCs from sheep inoculated with AdDsRed (black), AdOX40L (red), or AdCD70 (blue) and OVA were isolated at the different showed time points and cultured for 48 h on OVA-coated plates. The number of anti-OVA IgG-producing B cells was evaluated by serial dilutions in an ELISPOT assay. Results are expressed as the number of antigen-specific cells (ASCs) per 10^6^ cells in each treatment group. A positive control of PBMCs activated with 25 μg/ml LPS (Sigma) was always included to validate the ELISPOT assay. **p < 0.005 Two-way ANOVA with Dunnett’s post-test. The black arrow (3MD0) denotes the OVA booster in all animals.

To determine the contribution of the costimulatory molecules *Oa*OX40L and *Oa*CD70 to the humoral immunity induced to the classical antigen OVA, three sheep groups (n=7) were inoculated and bled as described in M&M with human recombinant adenovirus (Ad) expressing either of these two molecules (AdOX40L or AdCD70) or none (AdDsRed) concomitantly with OVA antigen. OVA-specific IgGs production was measured in the sera of inoculated sheep at days 0 (D0), 15 (D15), 30 (D30) and 90 (3MD0) post first inoculation (pi) and at days 7 (3MD7) and 30 (3MD30) post OVA booster (pb). Anti-OVA IgGs were detected after the first inoculation at D15 and D30 in all groups with higher values in the costimulatory inoculated groups than in the control group with statistically significant differences between the *Oa*CD70 costimulated group and control sheep at D30 (**Figure 3A**). Three months after the immunization anti-OVA IgGs values were not detectable in control group whereas low antibody levels were maintained in both costimulated sheep groups, with significant differences between the *Oa*CD70 inoculated group and control (**Figure 3A**). Higher levels in anti-OVA IgGs, at days 3MD7 and 3MD30, compared with those obtained after the first inoculation were detected after the booster inoculation in all groups, reaching the highest values at day 3MD7. At this timepoint, similar values in control and *Oa*OX40L or *Oa*CD70 costimulated groups were found. The IgGs values decreased by day 3MD30, but persisted above the values of the first immunization with slightly higher amounts in the *Oa*CD70 inoculated group (**Figure 3A**). We also assessed the presence of anti-OVA antibody secreting B cells at the different timepoints (**Figure 3B**). Circulating anti-OVA B cells could be detected in all groups from day 15 post-immunization and persisted throughout the experiment. Their numbers increased in the circulation after the booster inoculation in all three groups at day 3MD7, and similarly to anti-OVA IgG levels, subsequently decreased at day 3MD30. Importantly, the decrease in these anti-OVA-specific Ab-secreting B cells was reduced in the *Oa*CD70 costimulated group when compared to control, as significantly higher anti-OVA B cell numbers were detected in this costimulated group at this late timepoint (**Figure 3B**). Taken together, these data indicate that *Oa*CD70 delivery with an immunizing antigen could improve antibody titers to the antigen and survival of antigen specific B-cells.

### *Oa*CD70 improves T cell responses against OVA

**Figure 4:**
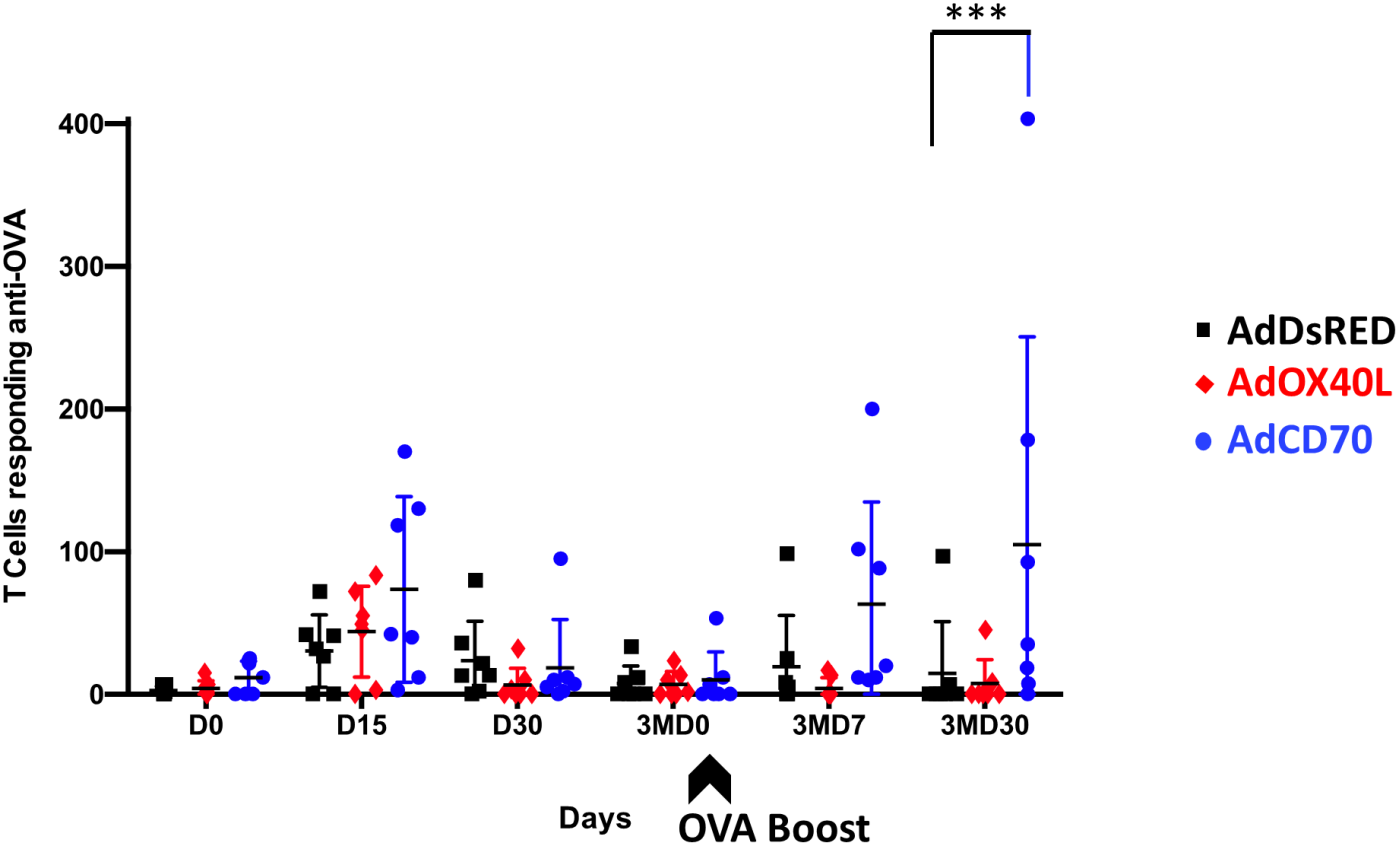
Specific IFN-γ production to OVA in PBMCs from the three groups of inoculated sheep detected by ELISPOT assay. PBMCs from sheep inoculated with control vector AdDsRed (black), AdOX40L (red), or AdCD70 (blue) were isolated at different times (x axes) and cultured for 48 h in the presence of OVA. The production of IFN-γ was measured using an ELISPOT assay. Data are presented as average (± SEM) IFN-γ spots above background for 10^6^ cells in each treatment group. A positive control of PBMCs activated with 1.25 μg/ml Con-A (Sigma) was always included to validate the ELISPOT assay. ***p < 0.001 Two-way ANOVA with Dunnett’s post-test. The black arrow (3MD0) denotes the OVA booster in all animals.

We next evaluated the effect of *Oa*OX40L and *Oa*CD70 molecules on the OVA-specific T-cell responses in sheep. PBMCs from immunized sheep obtained at the different timepoints were tested for IFN-γ production measured in ELISPOT assays (**Figure 4**). Specific IFN-γ production to OVA was detected at D15 post immunization in all sheep with higher average counts in costimulated sheep groups (red *Oa*OX40L: mean number of IFN-γ spot forming cells (SFC) for 10^6^ PBMC=43,92; blue *Oa*CD70: mean number of IFN-γ SFC/10^6^ PBMC=73,57) than in the control group (black DsRed: mean number of IFN-γ SFC/10^6^ PBMC =30,43). Interestingly, the AdCD70 inoculated group showed the highest numbers of IFN-γ producing T-cells on days D15, 3MD7 with statistically significant differences as compared to the control group at day 3MD30 (**Figure 4**).

**Figure 5:**
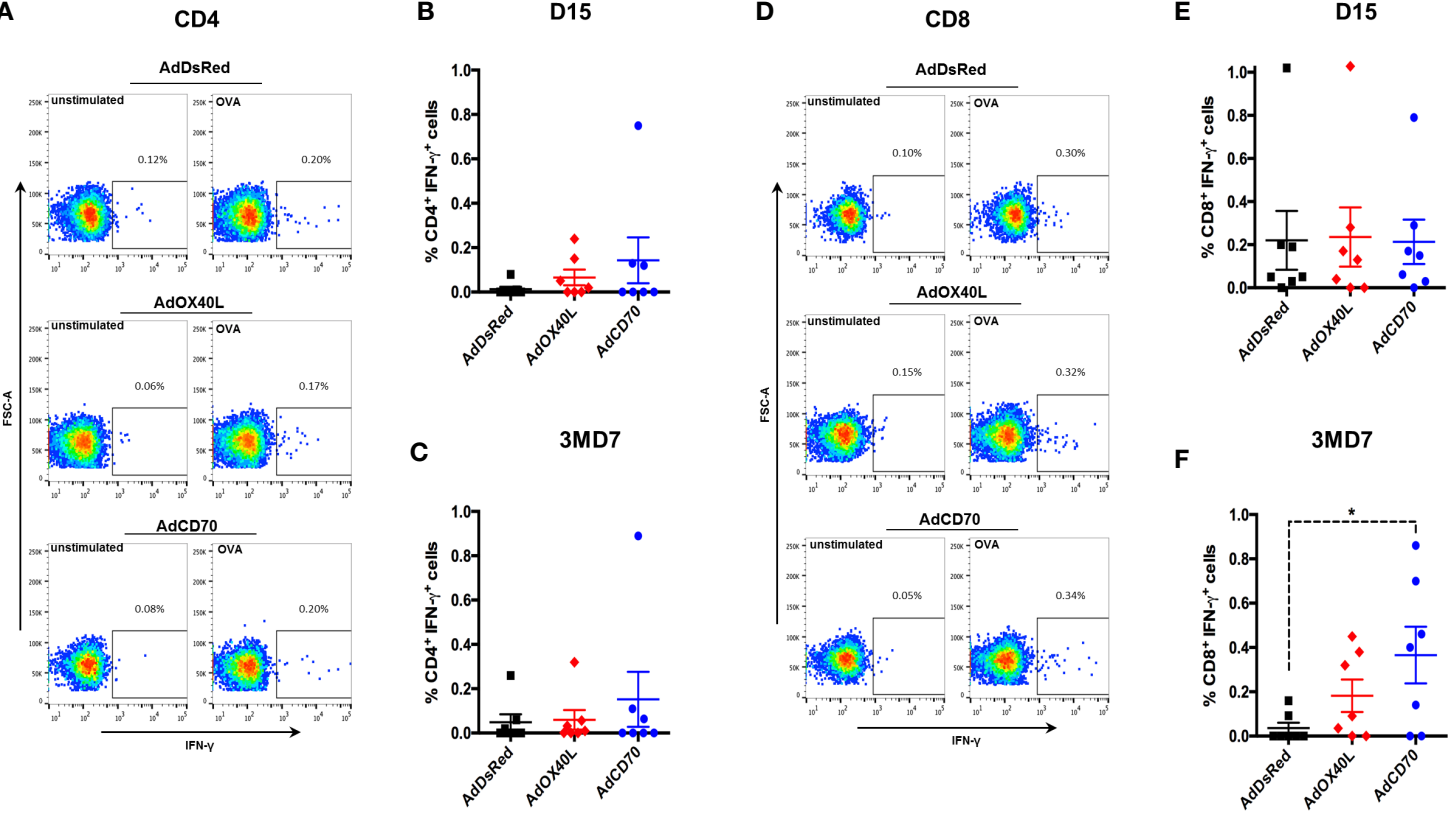
AdCD70 increases CD8^+^ T cell recall responses to OVA. PBMC were stimulated with OVA or left unstimulated as control for 6 hours. Brefeldin-A was added in the last 3 hours of incubation to block IFN-γ secretion. PBMC were surface stained with anti-CD4 and anti-CD8 antibodies, fixed and permeabilized and subsequently stained for intracellular IFN-γ. Fixable live-dead NIR marker was used to exclude dead cell from the analysis. **(A)** Representative dot-plots for CD4^+^ T cell IFN-γ production in control (unstimulated) and OVA-stimulated in AdDsRed, AdOX40L or AdCD70 inoculated sheep at day 15 post-immunization with OVA. Mean ( ± SEM) net IFN-γ production at **(B)** day 15 post-immunization and **(C)** day 7 post-OVA boost (3MD7) in CD4^+^ T cells. **(D)** Representative dot-plots for CD8^+^ T cell IFN-γ production in control (unstimulated) and OVA-stimulated in AdDsRed, AdOX40L or AdCD70 inoculated sheep at day 15 post-immunization with OVA. Mean ( ± SEM) net IFN-γ production at **(E)** day 15 post-immunization and **(F)** day 7 post-OVA boost (3MD7) in CD8^+^ T cells. * p < 0.05; (One-way ANOVA with Fisher’s LSD post-test).

To further characterize the T cell response to OVA, we performed intracellular IFN-γ staining for antigen-specific CD4^+^ and CD8^+^ T cells at day 15 post-immunization (D15) and at day 7 post-booster (3MD7) (**Figure 5**). Low frequency of OVA-specific IFN-γ-producing CD4^+^ T cells was detected in all groups at both timepoints with no significant differences between groups (**Figures 5A-C**). No differences between groups in the percentage of OVA-specific IFN-γ-producing CD8^+^ T cells were detected at day 15 post-immunization (**Figures 5D, E**). Nonetheless, 7 days after the booster vaccination, the frequency of OVA-specific IFN-γ-producing CD8^+^ T cells statistically increased in the *Oa*CD70 costimulated group when compared to the AdDsRed group. The frequency of these cells was also slightly increased in the *Oa*OX40L group although this did not reach statistical significance. These data indicate that *Oa*CD70 could improve the reactivation of CD8^+^ T cells.

**Figure 6:**
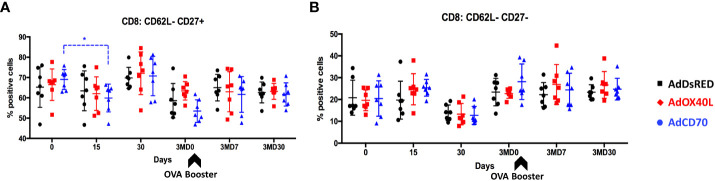
AdCD70 administration increases CD8^+^ T effector cells at day 15 post-inoculation. AdDsRed, AdOX40L or AdCD70 were administered at the time of immunization with OVA in three different groups of sheep. PBMC were obtained at different timepoints: day 0 (immunization), D15, D30, 3MD0 (prior to booster inoculation with OVA), 3MD7 (i.e. 7 days post-booster), and 3MD30 (i.e. 30 days post-booster); and percentages of **(A)** CD8^+^: CD62L^-^CD27^+^ and **(B)** CD8^+^: CD62L^-^CD27^-^ cells in PBMC evaluated by flow cytometry. Arrowheads denote OVA booster inoculations at day 90 (3MD0). * p < 0.05; (two-way ANOVA with Dunnett’s post test).

We also assessed the activation status of CD4^+^ and CD8^+^ T cells over time. To this end, the expression of the activation marker CD62L (which is lost upon activation) and the activation/memory marker CD27 (which is expressed on naïve, memory cells and in early activation, but downregulated on effector T cells) was analyzed in CD4^+^ and CD8^+^ T cells obtained from OVA immunized sheep at day 0, 15, 30, 3MD0 (pre-booster with OVA), 3MD7 (7 days post booster), and 3MD30 (30 days post booster) for each group. A significant reduction in CD8^+^ CD62L^-^ CD27^+^ cells was detected in the *Oa*CD70 group at day 15 when compared to day 0 (**Figure 6A**). Concomitant to this reduction, an increase in CD8^+^ CD62L^-^ CD27^-^ cells (**Figure 6B**) was detected although this did not reach statistical significance. These changes suggest that AdCD70 induces an increase in effector CD8^+^ T cells (CD8^+^ CD62L^-^ CD27^-^) which results in decreased number of early activated cells (CD8^+^ CD62L^-^ CD27^+^). No significant differences were detected in activated CD4^+^ cells, nor in CD8^+^ CD62L^+^ cells (**Supplementary Figure 3**). Taken together, these data indicate that *Oa*CD70 delivery with OVA could improve CD8^+^ T cell effector differentiation and recall responses.

### Cytokine expression profile after costimulation with *Oa*OX40L or *Oa*CD70

**Figure 7:**
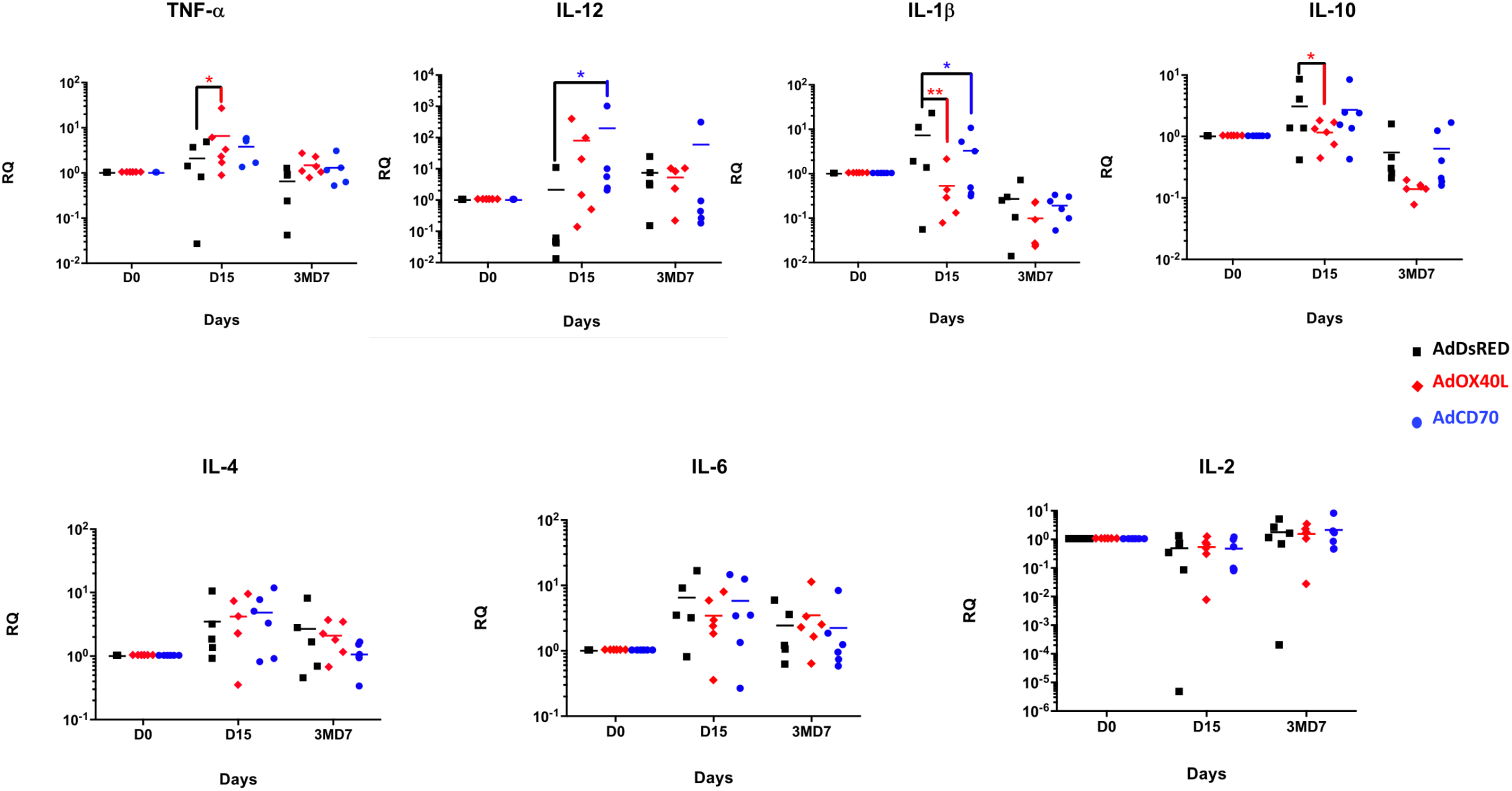
Analysis of expression of cytokine genes by qRT-PCR in sheep PBMCs. Cytokine gene expression from total RNA isolated from PBMCs obtained at days D15 and 3MD7 from the three sheep groups was measured by quantitative real-time PCR (qRT-PCR). Each individual sample was analyzed in duplicate. Relative levels of expression of cytokine mRNA were determined by comparative threshold cycle (*CT*) analysis, utilizing β-actin mRNA expression (internal control) as a reference. Statistical analysis was performed by two-way ANOVA test: *p < 0.05. Relative RNA quantity (RQ) = 2 ^-ΔΔ^ *^CT^*.

To broaden the analysis of the functionality of these molecules and their effects in the immune response, we assessed the induction of several cytokines by qRT-PCR in PBMCs obtained from the three groups of sheep at days 0, 15 and 3MD7 (**Figure 7**). No differences were observed between groups at 3MD7. At day 15 pi, sheep inoculated with *Oa*OX40L overexpressed TNF-α with statistical significance compared with DsRed inoculated sheep, while the expression of IL-12 was also increased although it did not reach statistical significance. Sheep inoculated with *Oa*CD70 showed a significant increase in IL-12 compared to control sheep at D15. Both adjuvant molecules induced a statistically significant decrease of IL-1β at D15 post-inoculation. IL-10 showed a significant decrease in *Oa*OX40L group of sheep at D15. All cytokines analyzed experimented a decrease from D15 to D7 after booster (3MD7) with the exception of IL-2, that slightly increased from D15 to day 3MD7 in all groups. This increase in IL-2 in the *Oa*CD70 group could be linked to the increased anti-OVA T cell activity detected at this timepoint (**Figure 5**). Overall it appears that *Oa*OX40L an *Oa*CD70 administration can influence the expressed cytokine profile of PBMC, with an increase in some proinflammatory cytokines such as TNF-α, or the Th1 cytokine IL-12.

## Discussion

Immunomodulators have the potential of boosting host immune responses thereby increasing the effectiveness of vaccines. The inclusion of immunomodulators in vaccine formulations can be an attractive approach in the case of suboptimal vaccinations. In here we report the generation of non-replicative recombinant human adenovirus expressing *Oa*OX40L or *Oa*CD70 to be used as immunomodulators. Prior work in our laboratory showed that the costimulatory signaling axes represented by OX40L-OX40 and CD70-CD27 are conserved and functional in the ovine species and that the recombinant ligands obtained can effectively elicit biological activity (Rojas et al., 2020). In this report we evaluated whether targeting these molecules *in vivo* in sheep could develop quality B and T cell responses to vaccination.

*In vivo* manipulation of costimulatory pathways should be done with care, as exemplified with the phase 1 clinical trial using a superagonist anti-CD28 monoclonal antibody that triggered a cytokine storm in volunteers (Suntharalingam et al., 2006). Unlike the CD28 pathway, which is central to T cell activation, the OX40/OX40L and CD27/CD70 axes are rather involved in the modulation of T and B cell responses and in the development of memory cells. It is therefore unlikely that their manipulation would trigger systemic responses. Indeed, we found *in vitro* that *Oa*OX40L and *Oa*CD70 only enhanced CD4^+^ and CD8^+^ T cell IFN-γ induction in response to mitogen ConA activation (Rojas et al., 2020). The data from the present *in vivo* experiments confirm that *Oa*OX40L and *Oa*CD70 delivery through recombinant adenoviral vectors does not trigger acute toxicity events in sheep, as no adverse effects were observed upon administration and PBMC counts were not significantly altered throughout the experiment. It should be nonetheless noted that these ligands designed to interact with *Oa*OX40 or *Oa*CD27 may plausibly exert additional biological effects through reducing target availability, and thereby preventing reverse signaling through *Oa*OX40L or *Oa*CD70.

An increase in monocytes (CD14^+^) was detected 30 days after the combined *Oa*OX40L and OVA inoculation and 7 days after OVA booster (**Figure 1**). The OX40/OX40L axis is known to affect the monocyte-T cell dialogue (Sun et al., 2018). Soluble administration of OX40 promotes inflammatory monocyte infiltration in a model of inflammatory liver disease. A reverse effect was observed in OX40-deficient mice, indicating that the interaction between OX40 on activated T cells and OX40L on monocytes was central to the inflammatory disease (Sun et al., 2018). Thus, OX40L administration could potentially promote indirectly the expansion of inflammatory monocytes by increasing the number of OX40-expressing T cells. In our case we only detected CD14^+^ cell expansion 30 days after antigen exposure and 7 days upon re-exposure. We also detected NK cell expansion upon *Oa*OX40L administration 30 days post booster inoculation. It is unlikely that at these late timepoints the expression of recombinant *Oa*OX40L is still present in the animals (Whittington et al., 1998). These late effects on monocyte and NK cell numbers could be an indirect effect of *Oa*OX40L administration and indicate the contribution of these cells in the contraction phase of adaptive immunity once the antigen is cleared (Pallmer and Oxenius, 2016; Gordon and Plüddemann, 2018). Overall, *Oa*OX40L or *Oa*CD70 delivery through adenovirus appears to be a safe approach to target these signaling axes *in vivo*. It would be nonetheless interesting in future work to assess the effect of these costimulatory molecules on innate immunity.

OX40 is known to control the number of effector T cells during primary or secondary immune responses as well as the frequency of memory cells that are generated [reviewed in (Weinberg et al., 2004; Croft, 2010)]. Due to the relevance of these features for vaccination, OX40 ligation has been proposed as a potent adjuvant strategy. For example OX40 was found to be critical for the development of CD8^+^ T cell responses against dominant and subdominant epitopes as well as for the generation of memory cells in vaccinia virus infection in mice (Salek-Ardakani et al., 2008). Furthermore, activation of OX40 signaling by an agonist antibody during peptide vaccination against vaccinia virus provided enhanced effector and memory cell activity, allowing for complete protection against a lethal respiratory dose of virus (Salek-Ardakani et al., 2011). We detected a slight increase in CD8^+^T cells response 7 days post-boost in *Oa*OX40L inoculated sheep when compared to control, which could indicate that in some individuals *Oa*OX40L administration improved the functionality of memory anti-OVA CD8^+^ T cells. Further work is nonetheless required to confirm this observation.

More notably, *Oa*OX40L altered the cytokine profile expressed by PBMC at day 15pi when compared to the control group. The increased TNF-α and IL-12 expression after *Oa*OX40L administration indicates the activation of a Th1 immune response (**Figure 7**). This function has been attributed to the OX40/OX40L interaction in several reports (Arestides et al., 2002; Fu et al., 2020; Gajdasik et al., 2020). Coincident with the increase in this Th1 polarizing cytokines, we also detected a statistical reduction in the levels of the regulatory cytokine IL-10 (Rojas et al., 2017a) which could indicate that *Oa*OX40L administration is promoting the development of a productive immune response. Further work will be required to clarify these findings.

Several hypotheses could explain the moderate immunomodulatory effects of *Oa*OX40L on the OVA response. One hypothesis is that OX40 actions are bypassed due to the signal provided to the TCR by the OVA antigen. The magnitude of the antigenic insult affects the signaling through OX40 (Gramaglia et al., 2000), and OVA is a potent immunogen. OX40 ligation may therefore not be critical to the development of adequate adaptive immune responses to OVA. The use of suboptimal antigens might be a better approach to detect differences in immune responses by OX40L costimulation pathways. Alternatively, the limited impact of *Oa*OX40L administration could be due to the fact that OX40 expression on T cells requires antigen recognition. Thus, simultaneous administration of *Oa*OX40L with antigen may be not ideal to optimally activate this costimulatory pathway. Further work will be required to define more precisely the *in vivo* impact of *Oa*OX40L administration on adaptive immunity.

The TNFR-TNF pair CD27-CD70 has similar costimulatory functions to the OX40-OX40L pairing (Ohshima et al., 1997; Krause et al., 2009). In humans, CD70 is able to induce the *in vitro* proliferation and cytokine production of αβ- and γδ- T cells (deBarros et al., 2011; Polak et al., 2012; Mahnke et al., 2013; Ramakrishna et al., 2015). For instance, CD27 engagement can drive CD8^+^ T-cell activation in the skin in the absence of CD4^+^ T cells help (Polak et al., 2012), probably due to its constitutive expression in naïve T cells. Our data indicated that *Oa*CD70 costimulation improves primary and recall responses to OVA. The highest IFN-γ values were detected in this group, showing statistically significant memory response activation at day 30 post-boost (**Figure 4**). The nature of these IFN-γ secretory cells is mainly CD8^+^ T cell (**Figure 5**). We also detected an increase in effector CD8^+^ T cells at day 15pi (**Figure 6**), which could suggest that *Oa*CD70 is promoting the full differentiation of these cells. It therefore appears that, in line with its function in other species, *Oa*CD70 can improve *in vivo* CD8^+^ T cell responses. We did however not detect significant effects in the CD4^+^ T cell compartment using OVA as antigen. The effects of *Oa*CD70 on this T cell compartment may be masked by the high immunogenicity of OVA.

In human, CD27 is also expressed on memory B cells and its ligation on B cells by CD70 is responsible, among other things, for plasma cell differentiation (Klein et al., 1998; Jacquot, 2000). *Oa*CD70 costimulation led to increased and prolonged antibody titers to OVA (**Figure 3**). It also appears that B cells were more readily activated upon re-exposure to the antigen. *Oa*CD70 administration has therefore the potential to improve antibody responses to antigens. The next step would be to determine whether antibody functionalities, such as neutralization antibody titers, can be improved by *Oa*CD70 administration.

*Oa*CD70 administration also altered the cytokine response of PBMC to OVA at day 15pi. We detected an increase in the Th1-promoting cytokine IL-12 upon *Oa*CD70 costimulation compared to the control group at day 15pi (**Figure 7B**). Although other authors have detected that signaling through CD27 causes an increase in IL-2 expression (Matter et al., 2008), the values in sheep stimulated with *Oa*CD70 were not significant at day 15pi. This could be due to the time of analysis since increase in IL-2 mRNA is typically detected within 4 hours of CD27-CD70 interaction (Peperzak et al., 2010). We nonetheless detected a slight increase in IL-2 levels at day 7pb in the *Oa*CD70 group, which coincided with the increased CD8^+^ T cell response to OVA. This increase in IL-2 could reflect the reactivation of CD8^+^ memory cells (CD27^+^ and CD45RA^-^) (Mahnke et al., 2013) at the time analyzed. The downregulation in *Oa*CD70-costimulated sheep of the pro-inflammatory cytokine IL-1β (**Figure 7C**), mainly produced by monocytes, dendritic cells and tissue macrophages (Garlanda et al., 2013), when compared to AdDsRed inoculated sheep, could restrict its downstream signaling into the NF-κB-mediated transcription of pro-inflammatory cytokines, and could ultimately limit inflammation initiation (Tulotta and Ottewell, 2018). Overall, it appears that *Oa*CD70 administration triggers a complex network of cytokine production in PBMC which will require further work to fully characterize.

Our data indicate that *Oa*CD70 delivery with the immunizing antigen could improve antibody titers to the antigen and prolong the presence of antibodies in the periphery. Moreover, *Oa*CD70 improved CD8^+^ T cell recall responses to the antigen and increased in PBMC the expression of the Th1-polarizing cytokine IL-12. *Oa*CD70 concomitant delivery with the immunizing antigen thus appears to improve cellular and humoral immunity. To develop effective vaccines against pathogens a combination of immunoglobulins and long-lasting memory CD8^+^ T cells should be generated. Receptors and ligands of the TNF family play important roles in controlling lymphocyte activation and survival during viral immunity. Therefore, activation of these costimulatory pathways could prove attractive in veterinary medicine. The present work furthers our understanding of the OX40-OX40L and CD27-CD70 pairings in sheep. Activation of these costimulatory pathways using a recombinant adenovirus as delivery system did not produce acute toxicity *in vivo* and improved to some extent adaptive immunity to the model antigen OVA. Further work is nonetheless required to dissect the effects of these molecules in sheep. Ultimately the use of costimulatory signals provided by these TNF-TNFR could help improve vaccination protocols.

## Data availability statement

The original contributions presented in the study are included in the article/**Supplementary Material**. Further inquiries can be directed to the corresponding author.

## Ethics statement

The animal study was reviewed and approved by with the recommendations in the guidelines of the Code for Methods and Welfare Considerations in Behavioural Research with Animals (Directive86/609EC; RD1201/2005) and all efforts were made to minimize suffering. Experiments were approved by the Committee on the Ethics of Animal Experiments (CEEA) (Permit number: 10/142792.9/12) of the Spanish Instituto Nacional de Investigación y Tecnología Agraria y Alimentaria (INIA) and the Comisión de Ética Estatal de Bienestar Animal (Permit numbers: CBS2012/06 and PROEX 228/14). Written informed consent was obtained from the owners for the participation of their animals in this study.

## Author contributions

JR, NS, and VM designed the experiments. VM directed the work. CM, JR, VM, and SM collected the samples at the experimental farm. DR-M, MA, AL-L, JR, and VM performed the experiments. NS, JR, and VM wrote the manuscript. All authors contributed to the article, read and approved the submitted version.

## Funding

This work was funded by AGL2015-64290R, RTI2018-094616-B-100 and PID2021-124872OB-I00 from the Ministerio de Ciencia (Spain), grant S2018/BAA-4370-PLATESA2 from Comunidad de Madrid (Fondo Europeo de Desarrollo Regional, FEDER).

## Acknowledgments

The authors wish to thank Dr. Alejo for helpful discussions and all the members of the Experimental Farm “La Chimenea” (IMIDRA) for their invaluable help with the animals.

## Conflict of interest

The authors declare that the research was conducted in the absence of any commercial or financial relationships that could be construed as a potential conflict of interest.

## Publisher’s note

All claims expressed in this article are solely those of the authors and do not necessarily represent those of their affiliated organizations, or those of the publisher, the editors and the reviewers. Any product that may be evaluated in this article, or claim that may be made by its manufacturer, is not guaranteed or endorsed by the publisher.

## Supplementary material

Supplementary Figure 1 Gating strategy for IFN-γ determination in CD4+ and CD8+ T cells. PBMC were stimulated with OVA or unstimulated as control for 6 hours. Brefeldin-A was added in the last 3 hours of incubation to block IFN-γ secretion. PBMC were surface stained with anti-CD4 and anti-CD8 antibodies, fixed and permeabilized and subsequently stained for intracellular IFN-γ. Fixable live-dead NIR marker was used to exclude dead cell from the analysis. Lymphocyte gating was performed using FSC-A and SSC-A parameters, and doublet events excluded using FSC-A and FSC-H. Live-dead NIR staining was used to exclude dead cells. CD4^+^ and CD8^+^ event gating was performed on live cells, and IFN-γ^+^ events were determined within these gates. Appropriate isotype and fluorescence minus one channel controls were performed for gate setting.

Supplementary Figure 2 AdOX40 and AdCD70 administration does not trigger unspecific CD4+, CD8+, WC1+ and B cells stimulation. AdDsRed, AdOX40L or AdCD70 were administered at the time of immunization with OVA. PBMC were obtained at different timepoints: day 0 (D0) (immunization), D15, D30, 3MD0 (prior to booster inoculation with OVA), 3MD7 (i.e. 7 days post-booster), and 3MD30 (i.e. 30 days post-booster); and percentages of **(A)** CD4^+^, **(B)** CD8^+^, **(C)** WC1^+^, **(D)** B cell marker^+^, cells in PBMC evaluated by flow cytometry. Arrowheads denote OVA booster inoculations at day 90 (3MD0). No statistical differences in cell percentages in PBMC between recombinant adenovirus administration regimes were detected when compared within the same day (two-way ANOVA with Dunnett’s post test).

Supplementary Figure 3 AdOX40L and AdCD70 administration did not trigger unspecific CD4+ or CD8+ T cell activation. Three groups of sheep were immunized with OVA, as a model antigen, and received concomittantly an inoculation of AdDsRed (as control), AdOX40L or AdCD70. The expression of the activation marker CD62L (which is lost upon activation) and the activation/memory marker CD27 (which is expressed on naïve, memory cells and in early activation, but downregulated on effector T cells) was analyzed in **(A-D**) CD4^+^ and **(E-F**) CD8^+^ T cells obtained from OVA immunized sheep at day 0, 15, 30, 90 (pre-booster with OVA), 3MD7 (7 days post booster), and 3MD30 (30 days post booster) for each group.
