# Supplementary figures and images for: Adenoviral delivery of soluble ovine OX40L or CD70 costimulatory molecules improves adaptive immune responses to a model antigen in sheep
Source: Front Cell Infect Microbiol. 2022 Sep 23;12:1010873. doi: 10.3389/fcimb.2022.1010873 (PMC9538494; doi:10.3389/fcimb.2022.1010873)

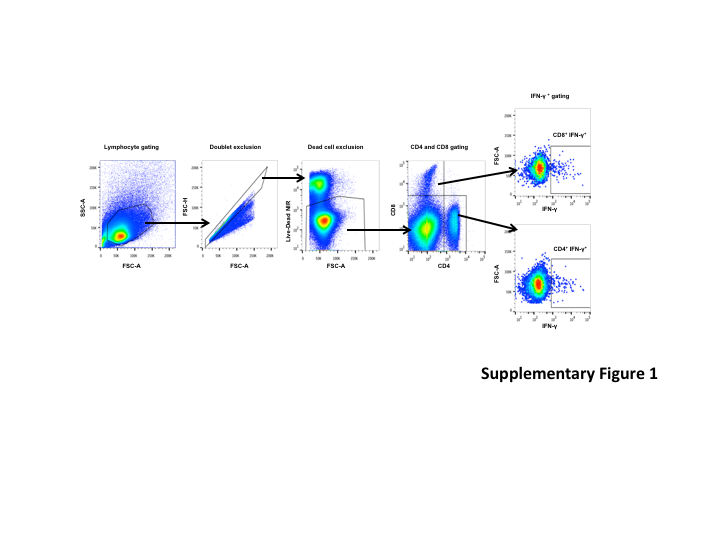

Supplement: Supplementary Figure 1 — Gating strategy for IFN-γ determination in CD4+ and CD8+ T cells. PBMC were stimulated with OVA or unstimulated as control for 6 hours. Brefeldin-A was added in the last 3 hours of incubation to block IFN-γ secretion. PBMC were surface stained with anti-CD4 and anti-CD8 antibodies, fixed and permeabilized and subsequently stained for intracellular IFN-γ. Fixable live-dead NIR marker was used to exclude dead cell from the analysis. Lymphocyte gating was performed using FSC-A and SSC-A parameters, and doublet events excluded using FSC-A and FSC-H. Live-dead NIR staining was used to exclude dead cells. CD4+ and CD8+ event gating was performed on live cells, and IFN-γ+ events were determined within these gates. Appropriate isotype and fluorescence minus one channel controls were performed for gate setting. [file Image_1.tiff]

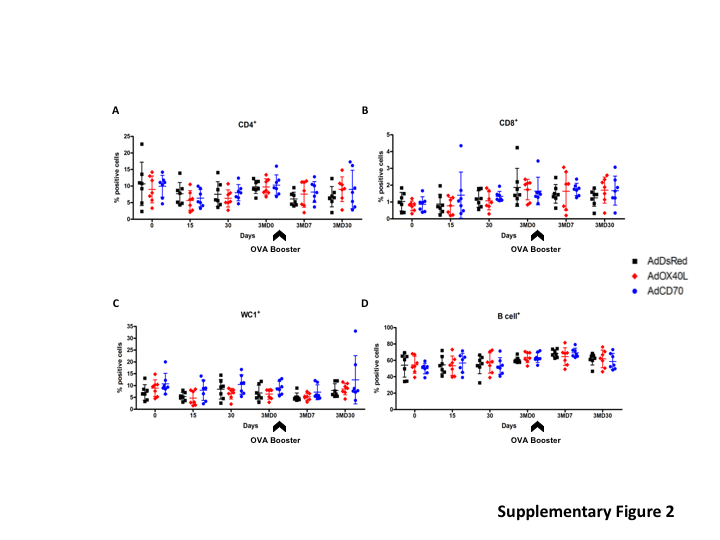

Supplement: Supplementary Figure 2 — AdOX40 and AdCD70 administration does not trigger unspecific CD4+, CD8+, WC1+ and B cells stimulation. AdDsRed, AdOX40L or AdCD70 were administered at the time of immunization with OVA. PBMC were obtained at different timepoints: day 0 (D0) (immunization), D15, D30, 3MD0 (prior to booster inoculation with OVA), 3MD7 (i.e. 7 days post-booster), and 3MD30 (i.e. 30 days post-booster); and percentages of (A) CD4+, (B) CD8+, (C) WC1+, (D) B cell marker+, cells in PBMC evaluated by flow cytometry. Arrowheads denote OVA booster inoculations at day 90 (3MD0). No statistical differences in cell percentages in PBMC between recombinant adenovirus administration regimes were detected when compared within the same day (two-way ANOVA with Dunnett’s post test). [file Image_2.tiff]

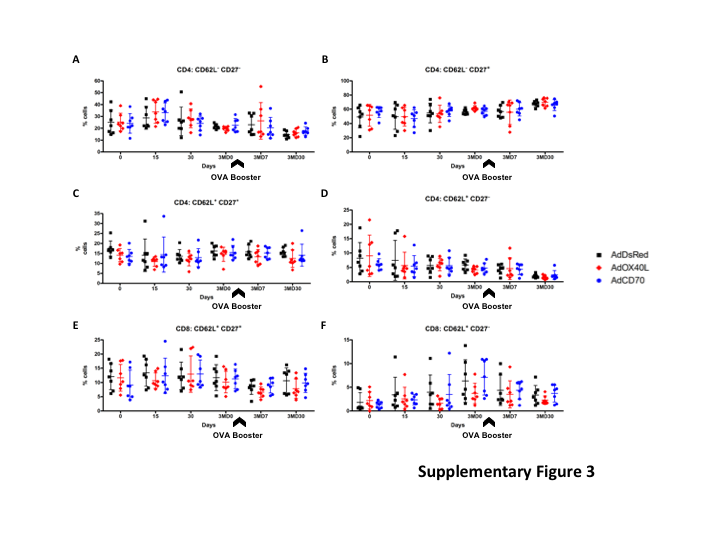

Supplement: Supplementary Figure 3 — AdOX40L and AdCD70 administration did not trigger unspecific CD4+ or CD8+ T cell activation. Three groups of sheep were immunized with OVA, as a model antigen, and received concomittantly an inoculation of AdDsRed (as control), AdOX40L or AdCD70. The expression of the activation marker CD62L (which is lost upon activation) and the activation/memory marker CD27 (which is expressed on naïve, memory cells and in early activation, but downregulated on effector T cells) was analyzed in (A-D) CD4+ and (E-F) CD8+ T cells obtained from OVA immunized sheep at day 0, 15, 30, 90 (pre-booster with OVA), 3MD7 (7 days post booster), and 3MD30 (30 days post booster) for each group. [file Image_3.tiff]
