# Supplementary material for: Adenoviral delivery of soluble ovine OX40L or CD70 costimulatory molecules improves adaptive immune responses to a model antigen in sheep
Source: Front Cell Infect Microbiol. 2022 Sep 23;12:1010873. doi: 10.3389/fcimb.2022.1010873 (PMC9538494; doi:10.3389/fcimb.2022.1010873)
Supplement: Supplementary file 4 [file Table_1.docx]

| **Primers: RNA Cytokine amplified** | **Secuences 5’-3’** |
| --- | --- |
| **TNF-α Forward** | CCAGAGGGAAGAGCAGTCC |
| **TNF-α Reverse** | GGCTACAACGTGGGCTACC |
| **IL-12 Forward** | CGTGTGGAAGCTGTGCACA |
| **IL-12 Reverse** | CTTTCCCTGGACCTGAACAC |
| **IL-1β Forward** | CGAACATGTCTTCCGTGATG |
| **IL-1β Reverse** | TCTCTGTCCTGGAGTTTGC |
| **IL-10 Forward** | CTTTAAGGGTTACCTGGGTTGC |
| **IL-10 Reverse** | CTCACTCATGGCTTTGTAGACAC |
| **IL-4 Forward** | CAGCATGGAGCTGCCT |
| **IL-4 Reverse** | ACAGAACAGGTCTTGCTTGC |
| **IL-6 Forward** | CCTCCAGGAACCCAGCTATG |
| **IL-6 Reverse** | GGAGACAGCGAGTGGACTGAA |
| **IL-2 Forward** | TCTACATGCCCAAGGTTAACG |
| **IL-2 Reverse** | CTACAGCCTTTACTGTCCC |
| **β-Actine Forward** | TGGGCATGGAATCCTG |
| **β-Actine Forward** | GGCGCGATGATCTTGAT |
|  |  |
| **RT Conditions** | 50ºC - 1h  70ºC - 5min  4ºC |
| **qPCR Conditions** | (95ºC - 10seg)  (55ºC - 10seg)  (72ºC - 10seg)  x40 cycles  95ºC - 10seg  65ºC - 60seg |
